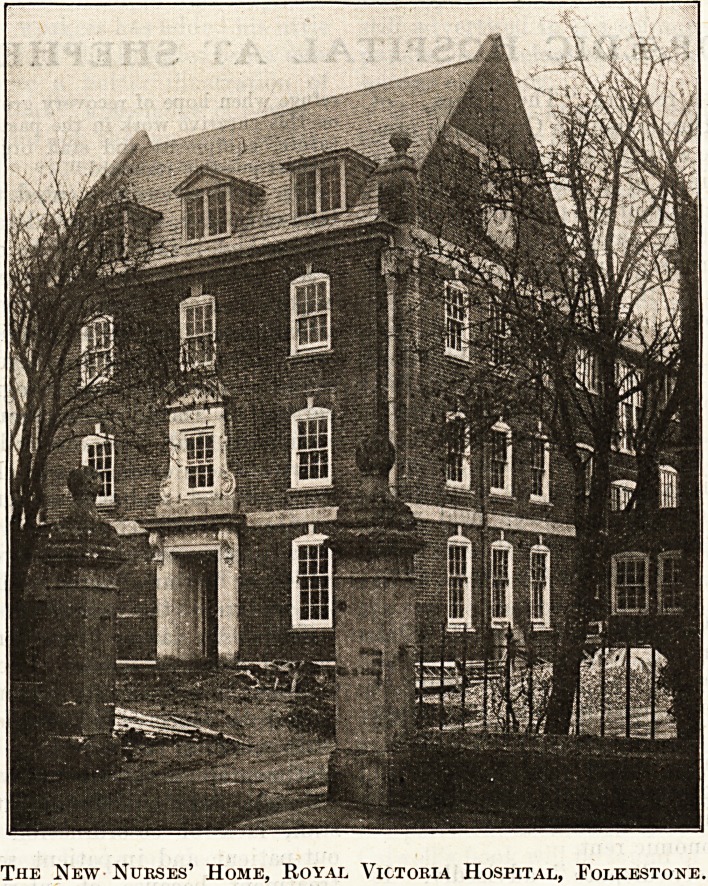# Health with Economy: The Position on the South Coast

**Published:** 1922-04

**Authors:** 


### HEALTH WITH ECONOMY.

### THE POSITION ON THE SOUTH COAST.—II. FOLKESTONE.

CONDITIONS at Folkestone are altogether different from those which we considered last month at the typical modern health resort of Eastbourne. The ancient town has to deal with two sets of problems, those of a health resort, and of a port with regular passenger services and a considerable food trade.

Folkestone is one of the ports scheduled under the Aliens Act, which requires medical inspection of all aliens landing and allowed to remain more than three months, or brought to the notice of the Medical Inspector by the Immigration Officers, who are under the Home Office. It is on the point of becoming a Port Sanitary Authority. The cross-Channel traffic is with Flushing, Boulogne and Calais. The food trade with Holland and France involves much work in connection with the Foreign Food Regulations; to mention only one item, 213,492 pork carcases were received from Holland during 1921, and as many as 2,500 may arrive on a single day. Last year 20,863 tons of food were received from Flushing, and 5,417 from Boulogne. In ordinary times vessels come in with timber and ice from the Baltic, and from the White Sea. The Channel Isles send stone and granite; the coastwise shipping brings timber from the Thames, and coal from the North; barges come from Southampton. Further assistance in the work of food inspection is desirable, but it is likely that the extra inspector's appointment will be deferred on account of the necessity for economy.

There is urgent need of a well-equipped cleansing and disinfecting station for the port, and it is hoped that, with the approval of the Ministry of Health, one may be installed this year. If so, it will be used when necessary for the civil population. Meanwhile, the work is being carried on at a temporary station half a mile from the harbour. There is an Isolation Hospital close by for contacts removed from infected ships. Smallpox is treated at the Smallpox Hospital, about 1½ miles from the pier. A motor ambulance is used for the removal of patients and their bedding.

Folkestone's water is drawn from deep wells in the chalk, capable of supplying between two and three million gallons a day. Further headings are in progress, and the tapping of a further supply is expected. The allowance per head works out at the very liberal figure of 50 to 60 gallons a day. The drought had not affected the town in any way until early this year, when as a measure of prudence it was decided to cut off the supply during the night. In this connection the Medical Officer of Health, Mr. M. G. Yunge-Bateman, considers that where waste of water is concerned, garages are among the worst offenders.

With the exception of some half-dozen houses on the outskirts of the town, which have cesspools, the water-carriage system of sewage disposal is general. At present the untreated sewage goes direct into the sea. There is in consequence, and has been for some time, pollution of the foreshore. A scheme has been approved by which the outfall will be extended on the eastward into deep water, and the question of screening is being considered. Chlorination is not thought practicable.

LIEUT.-COL. SIR STEPHEN PENFOLD, J.P.
(CHAIRMAN OF HEALTH COMMITTEE.)

One of the special needs of the town is a public abattoir, where proper control could be exercised, and a clearing house established for the inspection of meat arriving from outside the borough. The existing slaughter-houses, which could only be bought out at great expense, have many defects, not only from the hygienic and humanitarian standpoints, but because the cattle must be driven to them through narrow streets in populous neighbourhoods. Such as they are, they are kept in decent order.

Like every ancient town, Folkestone possesses certain districts in which the houses are in poor—sometimes decidedly unsatisfactory — condition. When permission can be obtained it will proceed with an improvement scheme, which began some fifteen to eighteen years ago with the building of about fifty houses. Under the Government Housing Scheme land was bought away from the town, on a south-ward-facing shelf of the Downs, for 250 houses. Of the 200 now sanctioned, about 160 have already been built and occupied. They are of several types, with four, three and two bedrooms, and include twenty tenements of two rooms. They form a pleasant little red-and-white garden city, well spread out, which will be still more agreeable to look at when its gardens are matured and its trees have grown.

One of Folkestone's special problems is that of the holiday children, whom well-meaning people in London send down into any houses that will receive them, without reference to the local sanitary authorities. In consequence, the Medical Officer of Health last year found several houses in the "unhealthy area" taking in children. These houses were already sufficiently, or more than sufficiently, filled, and the young visitors, stowed away perhaps three in a bed, were sometimes worse off than in the homes from which they came, especially in bad weather when they could not get out. In 1920 an outbreak of scarlet fever was distinctly traced to holiday children, sent down in an infective condition or from infected areas. It is much to be wished that the providers of holidays would consult the sanitary authorities, who could tell them beforehand whether the chosen houses and districts were suitable. An even better plan would be to organise camps for the youngsters.

Folkestone's infant welfare and school medical services are admirably housed. The centre is at the Harvey Grammar School, founded for twenty boys by a brother of the great William Harvey, a native of the town. Folkestone now has a large grammar school of nearly 300 boys, and was able to give up the old school building to an object, after all, not far from the benevolent intention of the founder. In the principal classroom mothers and children assemble, and here, in ample space, is the apparatus for remedial exercises. Dental and other minor operations are performed in a spacious room, hospital-like in its well-kept whiteness and brightness. The small patients are passed on for "cleaning up" to a communicating room fitted with lavatory basins, and thence to the "recovery room." Other apartments are allotted to ear, eye and throat troubles, and the medical attendants and clerks are well lodged. It may be mentioned that no sooner was this centre opened than the Folkestone rooks appropriately set up a nursery in the schoolyard elms!

Sir Stephen Penfold, the Chairman of the Health Committee, is also Chairman of the Royal Victoria Hospital, to which he kindly acted as our guide. The hospital is a dignified red-brick building, facing south towards the pretty Radnor Park, and commanding a fine view of the Downs. Its position is such that it loses nothing in the way of sun and air that the kindly south coast climate can give. Flooded with light on a sunny day of early spring, and with so spacious and exhilarating an outlook, it seemed a most desirable place to be ill in, and we heard without surprise that in such singularly favourable conditions patients do extremely well. Of its sixty beds, a daily average of 54 are occupied. A few tuberculosis cases are admitted, and live almost entirely on the balconies, which are a feature of this hospital. The V.D. Clinic is housed in a small detached building.

The New Nurses' Home, Royal Victoria Hospital, Folkestone.

The town is proud of its hospital, and supports, among other organisations for its help, a Ladies' Linen League, which at the time of our visit had just presented some £100 worth of sheets and napery. It was pleasant to learn from the Matron, Miss C. Browne, that a balcony and a lift were given by local schools.

The new Nurses' Home, to be opened in June, was in March at a stage when its coming perfections began to be apparent. It is being built by Messrs. D. Baker & Co., at a cost of £14,000; the architect is Mr. Dahl, F.R.I.B.A., of Messrs. Bromley & Dahl. Like Mr. William De Morgan's most charming heroine, it has not an angle anywhere in its disposition, and the result is something that fills the average householder with hopeless envy. Floor meets wainscoting, and ceiling meets wall, hospital-fashion, in a gentle curve; woodwork and plaster-work are alike innocent of foolish dirt-collecting mouldings. There are eleven bedrooms on each of two floors, with room for expansion in the attic story. Each nurse will have a separate bedroom; each bedroom has a fixed basin with hot and cold water, and a "splashboard" of mosaic, and in the thickness of the wall a roomy wardrobe cupboard. There is no picture-rail dust-trap, but a wooden frieze, flush with the wall, is provided, into which nails may be driven. This is worth record, for the picture-hanging instinct is part of the home-making instinct, and the nurse who can satisfy it is likely to be happier, and therefore more efficient, than she who is sternly denied such indulgences. The apartments of the night staff are specially arranged with a view to the cutting off of noises. Shower and ordinary baths are provided on each floor. The tea-making facilities are especially fascinating ; there is a cupboard for crockery next to a washing-up sink ; a gas-ring can be drawn out on to a shelf above the sink for kettle-boiling, and returned to the cupboard through an aperture provided for the purpose. This thoughtful and imaginative care for the details that collectively make comfort is evident in the nurses' big common-room and the sisters' sitting-rooms, which possess radiators for warmth and open grates for the delight of the eye and of British sentiment. A terrace on the shady side of the building, looking out to the Downs, furnishes an ideal spot for tea and leisure. The staff's grass lawn-tennis courts have vanished past restoration under the builders' dump, but hard courts are to take their place when the money can be found.

As shown in the accompanying photograph, the Home is connected on two levels with the main hospital block, from which the Massage and X-Ray departments will be transferred when the new basement is completed. On the migration of the nurses, the rooms that they now occupy are to be adapted as small wards for middle-class paying patients.

## Figures and Tables

**Figure f1:**
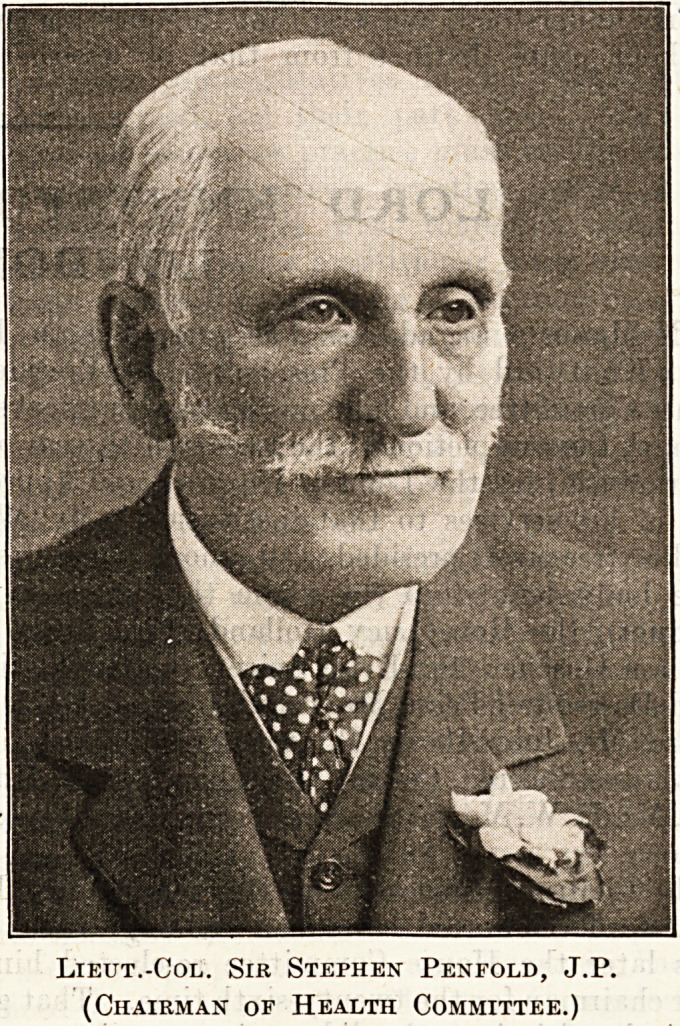


**Figure f2:**